# Sequential E2 levels not ovarian maximal diameter estimates were correlated with outcome of cetrotide therapy for management of women at high-risk of ovarian hyperstimulation syndrome: a randomized controlled study

**DOI:** 10.1186/s12905-017-0466-z

**Published:** 2017-11-13

**Authors:** Khalid M. Salama, Hesham M. Abo Ragab, Mohammed F. El Sherbiny, Ali A. Morsi, Ibrahim I. Souidan

**Affiliations:** Department of Obstetrics and Gynecology, Faculty of Medicine, Benha University, Benha, Egypt

**Keywords:** Ovarian hyper stimulation syndrome, Cetrotide therapy, Embryo freezing

## Abstract

**Background:**

Ovarian hyperstimulation syndrome (OHSS) is an important condition with considerable morbidity and a small risk of mortality and most commonly results as an iatrogenic condition following follicular stimulation of the ovaries**.** We aimed to evaluate safety and efficacy of 3-day cetrotide therapy started on day of oocyte retrieval (Day-0) in women at high-risk for development of ovarian hyperstimulation syndrome (OHSS) after GnRH agonist induction protocol.

**Methods:**

Forty-eight women fulfilling inclusion criteria underwent ultrasound scanning for maximal ovarian diameter (MOD) estimation and ascites grading. Patients underwent embryo freezing, but the study group received 3-day Cetrotide sc injection (0.25 mg/day) started on Day-0. Serum E2, pain scores and MOD were checked daily. Hematocrite value (Ht%), total leucocytic count (TLC), gastrointestinal (GI) manifestations and ascites grading were re-evaluated on Day-3, 6 and 8.

**Results:**

Sequential serum E2 levels decreased significantly in both groups with significantly lower levels in the study group. Sequential MOD estimates showed non-significant difference between the two groups and versus Day-0 estimates. On Day-2, pain scores showed progressive significant decrease compared to Day-0 scores in both groups with significantly lower scores in the study group. On Day-3; four control patients still had vomiting and by Day-6, 6 of the control patients still had GI manifestations with significant difference versus the study group. Compared to Day-0 estimates, Ht% and TLC were significantly lower on Day-3, 6 and 8 in the study group, but only on Day-8 in the control group. Day-3 and Day-8 ascites grading in both groups was significantly lower compared to respective Day-0 grading with significant difference in favor of the study group. Six patients required hospitalization, but without mortalities. Day-3 E2 levels in the study group showed positive significant correlation with clinical and other laboratory data and ascites grading, while the correlation was non-significant with MOD.

**Conclusion:**

The 3-day cetrotide therapy starting after oocyte retrieval with embryo transfer freezing could be an appropriate management policy for women received GnHR-agonist induction protocol and were at high-risk for OHSS. Sequential E2 serum levels could predict outcome more perfectly than sequential MOD estimates.

**Trial registration:**

Trial registration (clinicaltrial.gov registration) NCT02823080 (retrospective) Initial Release 21–6-2016 Last Release 3–1-2017 Unique Protocol ID: Benha U Secondary IDs: kmsalama.

## Background

Ovarian hyperstimulation syndrome (OHSS) is a disease state that occur secondary to ovarian stimulation in which ovaries were enlarged with fluid transudation into body spaces and subsequent reduction of circulatory volume that may predispose to increased risk of venous thrombosis and decreased organ perfusion [1]. Multiple predisposing factors were supposed for the possibility of developing OHSS during ovarian stimulation protocols. Age, antral follicles count, infertility cause, hypothyroidism and positive history of ovarian surgery were the most important predictors of OHSS, but the number of follicles and serum estradiol level on human chorionic gonadotropin day were the strongest predictive variables [2].

For prophylaxis against development of OHSS, multiple trials compared gonadotropin-releasing hormone (Gn-RH) agonist (GnRH-a) versus Gn-RH-antagonist (GnRH-ant) induction protocols wherein Kdous et al. [3] found GnRH-ant allows a higher flexibility in treatment, a lower dose of follicle stimulating hormone (FSH) was required and a shorter period of stimulation with no case of OHSS versus 3% with GnRH-a therapy. Martínez et al. [4] found stimulation protocol based on gonadotropins and a single dose of cetrorelix acetate 3 mg was adequate in terms of safety and Depalo et al. [5] also documented that protocols with GnRH-ant were effective in preventing a premature rise of LH and induced a shorter and more cost-effective ovarian stimulation compared to the long agonist protocol.

Various drugs were evaluated for the prevention or treatment of OHSS; experimentally, in rat model of OHSS, both myo-inositol and metformin reduced vascular endothelial growth factor (VEGF) and COX-2 expressions with decreased vascular permeability and blood E2 levels in the groups treated with myo-inositol or metformin compared to the OHSS group [6]. Another experimental animal trial reported that letrozole and cabergoline were equally effective to prevent OHSS, reducing the ovarian diameter, vascular permeability, pigment epithelium-derived factor (PEDF) and VEGF levels to similar extents [7]. Turan et al. [8] experimentally found prophylactic vitamin D supplementation effectively increases PEDF, but is not sufficiently effective in preventing OHSS. Clinically, Kılıç et al. [9] found prophylactic treatment with the dopamine agonist, cabergoline, reduced the incidence of OHSS in women at high risk undergoing IVF/ICSI treatment, but the effects of cabergoline on live birth, miscarriage, and congenital abnormalities are still uncertain.

The current prospective comparative study aimed to evaluate the efficacy of the 3-day cetrotide therapy starting on the day of oocyte retrieval in women at high-risk for development of OHSS after GnRH agonist induction protocol as judged by changes of serum E2 level and maximal ovarian diameter.

## Methods

The current study was conducted at Assisted Reproduction Unit at Almana general, Hospital, KSA and some private centers in Egypt. The study protocol was approved by the Local Ethical Committee. The study was started from October 2014 till August 2016. The study aimed to include women suspected to be at high risk for development of OHSS during agonist ovarian stimulation protocol. Inclusion criteria included the number of retrieved oocytes must be ≥20; mean number of follicles with a diameter of >16 mm was ≥18; serum E2 concentrations of ≥3500 pg/ml; ovarian diameter on the day of ovum retrieval of >10 cm; and presentation of evident symptoms of OHSS on the day of aspiration [10]. The study included only women of couples who singed written consent to participate in the study, to undergo embryo freezing and to postpone for transfer of cryo-preserved embryo. Patients followed GnRH-antagonist protocol, or who had liver, hepatic, cardiac or pulmonary dysfunction, coagulation disorders, or refused to sign the consent were excluded from the study.

All patients were clinically evaluated for the presence of abdominal pain and if present, it was graduated using a numerical pain visual analogue scale (VAS) with 0 means no pain and 10 means severe intolerable pain [11]. Patients were evaluated for the presence of nausea and/or vomiting; nausea was defined as the unpleasant sensation associated with awareness of the urge to vomit and was scored as 0 = nil, 1 = mild, 2 = moderate, and 3 = severe nausea. Vomiting was defined as the forceful expulsion of gastric contents from the mouth and was scored as 0 = nil, 1 = one mouthful, and 2 = more than one mouthful of vomitus [12]. Sense of abdominal distension was scored using verbal analogue scale as nil, mild, moderate, and severe distension. Blood samples were obtained under complete aseptic condition for estimation of serum E2 level and determination of hematocrit value (Ht%) and total leucocytic count (TLC).

Then, all patients underwent ultrasound scanning for estimation of ovarian measurements that were represented as the maximal ovarian diameter (MOD) and for ascites grading if present. Ultrasound scanning was performed using a 5 MHz vaginal probe (probe EC4 accuvix), otherwise a 3.5 MHz, 2.6 MHz or 5 MHz abdominal probe (probe C3-71 M accuvix) was used if visualization using a vaginal probe was compromised. Ascites was graded according to the quantity of fluid accumulation in the peritoneal cavity with the patient in the anti-Trendelenburg position according to Lainas et al. [13].

All women fulfilling inclusion criteria were graded, as regards the severity of OHSS manifestations, according to classification grading of OHSS proposed by Navot et al. [14]. All patients followed cancellation embryo replacement and embryo freezing protocol and were randomly allocated, using sealed envelops, into two equal groups. Control group included women received traditional symptomatic treatment including analgesics, antispasmodics and antiemetic therapy. Study group received, in addition to symptomatic treatment, cetrotide subcutaneous injection (cetrorelix acetate, Merk Serono, UK) in a daily dose of 0.25 mg according to Albano et al. [15] started on day of oocyte retrieval for 3 days.

All patients were managed as outpatients unless presented with severe manifestations necessitated hospital admission. Pain scores, serum E2 levels and MOD were evaluated daily. Patients were evaluated for associated symptoms previously determined during clinical evaluation, ascites grading and TLC and Ht value were evaluated at 3 and 6 days and on end of the trial on the 8th day after oocyte retrieval.

### Study outcome

1. Primary outcome was defined as occurrence and extent of change of elevated blood E2 levels and change of MOD.
2. Secondary outcome was defined as resolution or diminution of clinical, other laboratory and US manifestations determined prior to start of therapy.

### Statistical analysis

Obtained data were presented as mean ± SD, numbers and percentages. Results were analyzed using One-way ANOVA with post-hoc Tukey HSD Test and Chi-square test (X^2^ test). Statistical analysis was conducted using the SPSS (Version 15, 2006) for Windows statistical package. *P* value <0.05 was considered statistically significant.

## Results

**Fig. 1:**
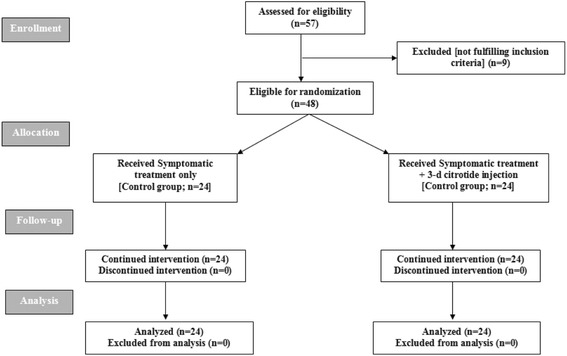
Consort Flow Sheet

Fifty-seven patients at high-risk for developing OHSS were assessed for eligibility; 9 patients were excluded for being not fulfilling inclusion criteria, while 48 patients were randomized into two groups (Fig. 1).

Patients of both groups showed non-significant (*p* > 0.05) differences concerning demographic, ovarian stimulation data and OHSS-related data as shown in Tables 1 and 2.

**Table 1:** Demographic and stimulation data of included patients

Data				Control	Study	*P* value
Age (years)	Strata	≤25		6 (25%)	11 (45.8%)	>0.05
		26–30		11 (45.8%)	8 (33.3%)	
		>30		7 (29.2%)	5 (20.9%)	
	Total			28.6 ± 3.3	27 ± 4	>0.05
BMI data	Weight (kg)			78 ± 6.1	80.5 ± 7.7	>0.05
	Height (cm)			168 ± 3.6	167 ± 4.2	>0.05
	BMI (kg/m^2^)	Strata	<25	6 (25%)	3 (12.5%)	>0.05
			25–30	14 (58.3%)	12 (50%)	
			>30	4 (16.7%)	9 (37.5%)	
		Total		27.6 ± 2.4	28.9 ± 2.9	>0.05
Duration of infertility	Strata	≤5		7 (29.2%)	8 (33.3%)	>0.05
		>5		17 (70.8%)	16 (66.7%)	
	Total			3.8 ± 2	4.2 ± 2.1	
Cause of infertility	Male factor			11 (45.8%)	10 (41.6%)	>0.05
	Female factor			5 (20.9%)	7 (29.2%)	
	Mixed			8 (33.3%)	7 (29.2%)	
PCOS	Yes			20 (83.3%)	19 (79.1%)	>0.05
	No			4 (16.7%)	5 (20.9%)	
Basal FSH (mIU/ml)				7.5 ± 1	7.31 ± 0.77	>0.05
Total gonadotrophin dose (IU/ml)				1605 ± 404	1475.4 ± 382	>0.05
Time of stimulation (days)				8.1 ± 1.4	8.2 ± 1.6	>0.05
HCG dose (IU/ml)				5800 ± 1500	5977 ± 1788	>0.05

Data are presented as mean ± SD & numbers; percentages are in parenthesis

**Table 2:** OHSS-related clinical and laboratory data

				Control	Study	*P* value
Number of retrieved oocytes				20.4 ± 1.6	21.1 ± 1.7	>0.05
Number of follicles (diameter of >16 mm)				17.1 ± 2.6	18 ± 3.2	>0.05
Serum E2 concentrations (pg/ml)				5004 ± 792	4761 ± 940	>0.05
Ovarian maximum diameter (mm)				10.4 ± 5.8	9.4 ± 4.5	>0.05
Frequency of associated symptoms		Distension		19 (79.2%)	20 (83.3%)	>0.05
		Nausea		19 (79.2%)	18 (75%)	>0.05
		Vomiting		10 (41.7%)	12 (50%)	>0.05
Laboratory findings	Ht value (%)	Strata	<40	14 (58.3%)	17 (70.8%)	>0.05
			40–45	6 (25%)	6 (25%)	
			>45	4 (16.7%)	1 (4.2%)	
		Mean value		39.8 ± 4.2	42.2 ± 5.2	>0.05
	TLC (× 10^3^ cells/ml)	Strata	<10	4 (16.7%)	12 (50%)	>0.05
			10–15	14 (58.3%)	8 (33.3%)	
			>15	6 (25%)	4 (16.7%)	
		Mean count		11.6 ± 3.7	12.75 ± 3.1	>0.05
Ascites	US detected	Low		6 (25%)	8 (33.3%)	>0.05
		Moderate		18 (75%)	16 (66.7%)	
	Marked (Clinically detected)			0	0	

Data are presented as mean ± SD & numbers; percentages are in parenthesis

**Fig. 2:**
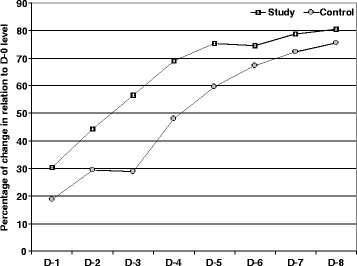
Mean estimated serum E2 levels during follow-up period

Throughout the observation period, estimated serum E2 levels showed progressive significant (*p* < 0.05) decline compared to Day-0 levels in patients of both groups with significantly (p < 0.05) lower serum E2 levels estimated in patients of study group compared to patients of control group. Moreover, the percentages of decrease of estimated serum E2 levels during the follow-up period were progressively increasing in both groups but were significantly higher in patients of study compared to patients of control group (Fig. 2). Interestingly, mean MOD showed gradual non-significant (*p* > 0.05) decrease in patients of both groups compared to Day-0 MOD (Table 3).

**Table 3:** Serum E2 levels and maximal ovarian diameter (MOD) of studied patients estimated throughout the observation period in both groups

	Serum E2 level (pg/ml)			Maximal ovarian diameter (mm)		
	Control	Study	*P* value	Control	Study	P value
Day-0	5004 ± 792	4761 ± 940	>0.05	10.4 ± 5.8	9.4 ± 4.5	>0.05
Day-1	4212 ± 950^a^	3397 ± 1361^a^	0.021	10.2 ± 5.6	9.3 ± 4.6	>0.05
Day-2	3620 ± 1037^a^	2772 ± 1456^a^	0.011	9.6 ± 4.6	9.1 ± 4.2	>0.05
Day-3	3592 ± 862^a^	2067 ± 900^a^	0.005	9.4 ± 4.9	8.7 ± 4.3	>0.05
Day-4	2570 ± 914^a^	1452 ± 617^a^	0.007	9.2 ± 4.7	8.5 ± 4.3	>0.05
Day-5	1914 ± 965^a^	1173 ± 180^a^	0.003	9 ± 4.6	7.8 ± 4	>0.05
Day-6	1544 ± 812^a^	1173 ± 180^a^	0.006	8.5 ± 4.6	7.5 ± 4.2	>0.05
Day-7	1275 ± 588^a^	969 ± 118^a^	0.001	8.2 ± 4.5	7.3 ± 4	>0.05
Day-8	1044 ± 200^a^	902 ± 66^a^	0.001	7.9 ± 4.2	7.1 ± 3.7	>0.05

Data are presented as mean (±SD); Day-0: time of enrolment; ^a^: significant difference versus respective Day-0 data; *P* value for the difference between both groups

Day-1 VAS pain scores were non-significantly (*p* > 0.05) decreased in both groups compared to Day-0 scores and to each other. In study group, on the 2nd day, pain started to be alleviated and scores showed progressive (*p* < 0.05) significant decrease compared to Day-0 scores and to control group. On contrary, pain scores of patients in control group were non-significantly decreased since Day-2 till Day-4, but significantly decreased thereafter till Day-8 in comparison to Day-0 scores (Table 4).

**Table 4:** Mean pain VAS score determined throughout the observation period in both groups

	Control	Study	*P* value
Day-0	5.4 ± 1.7	5.3 ± 1.6	>0.05
Day-1	5 ± 1.3	4.3 ± 1.3^a^	0.045
Day-2	4.9 ± 1.8	3.6 ± 1^a^	0.003
Day-3	4.6 ± 1.8	2.3 ± 1.8^a^	0.001
Day-4	4.5 ± 1.4	1.6 ± 1.2^a^	0.001
Day-5	3.7 ± 2^a^	0.92 ± 1.1^a^	0.001
Day-6	2.4 ± 1.1^a^	0.33 ± 0.6^a^	0.001
Day-7	1.6 ± 1.1^a^	0.17 ± 0.38^a^	0.001
Day-8	1.1 ± 1.7^a^	0.13 ± 0.34^a^	0.001

Data are presented as mean (±SD); Day-0: time of enrolment; ^a^: significant difference versus respective Day-0 data; *P* value for the difference between both groups

Concerning gastrointestinal (GI) manifestations; at Day-0, 37 patients had nausea, 22 patients had vomiting and 39 patients had abdominal distension of varying severity with non-significant (*p* > 0.05) difference between both groups. Nine patients had severe nausea and one of them had severe vomiting, while 5 patients had severe abdominal distension. On Day-3; 17 patients had nausea, 4 patients had vomiting and 24 patients still had abdominal distension; all of still complaining patients were of mild or moderate severity grades. As regards vomiting no patient in study group, while 4 patients in control group still had liability to vomit with significant (*p* < 0.05) difference in favor of study group. On contrary, the frequency of patients still had nausea and abdominal distension was non-significantly (*p* > 0.05) lower in study group compared to control group. By the 6th day, 6 patients in control group still had GI manifestations with significant (*p* < 0.05) difference versus study group. However, by the 8th day, all patients were free of GI manifestations (Table 5).

**Table 5:** Patients’ distribution among severity grades of GI manifestations determined at Day-3 and Day-6 compared to Day-0 distribution in both groups

Symptom	Time	Control				Study				*P* value
		Nil	Mild	Moderate	Severe	Nil	Mild	Moderate	Severe	
Nausea	Day-0	6	7	6	5	5	8	7	4	>0.05
	Day-3	12	8	4	0	19	4	1	0	>0.05
	Day-6	22	2	0	0	24	0	0	0	0.047
	Day-8	24	0	0	0	24	0	0	0	>0.05
Vomiting	Day-0	12	8	4	0	14	7	2	1	>0.05
	Day-3	20	4	0	0	24	0	0	0	0.037
	Day-6	24	0	0	0	24	0	0	0	>0.05
	Day-8	24	0	0	0	24	0	0	0	>0.05
Abdominal distension	Day-0	4	7	10	3	5	8	9	2	>0.05
	Day-3	9	12	3	0	15	8	1	0	>0.05
	Day-6	20	4	0	0	24	0	0	0	0.037
	Day-8	24	0	0	0	24	0	0	0	>0.05

Mean Ht value and TLC showed progressive decline throughout the observation period with non-significant (*p* > 0.05) difference between both groups. However, in comparison to Day-0 levels, estimated Ht value and TLC were significantly (*p* < 0.05) lower at Day-3, 6 and 8 in study group while in control group the difference was significant (*p* < 0.05) only in Day-8 estimates (Table 6).

**Table 6:** Mean Ht value and TLC estimated throughout the observation period in both groups

	Ht value (%)		TLC (10^3^/ml)	
	Control	Study	Control	Study
Day-0	39.8 ± 4.2	42.2 ± 5.2	11.6 ± 3.7	12.75 ± 3.1
Day-3	38.8 ± 3.3	38.7 ± 3.3^a^	11.3 ± 3.6	11.5 ± 2.8^a^
Day-6	38 ± 3.1	37.3 ± 2.5^a^	11.2 ± 3.6	11 ± 1.9^a^
Day-8	37.5 ± 1.5^a^	36.8 ± 2.6^a^	10.1 ± 2.2^a^	9.75 ± 3.1^a^

Data are presented as mean (±SD); ^a^: significant difference versus respective Day-0 data

On Day-0, no patient developed marked ascites, 34 patients had moderate and 14 patients had mild ascites with non-significant (*p* > 0.05) difference between both groups. On Day-3, three patients in control group developed marked (clinically detected) ascites, 14 patients had moderate and 31 patients had mild ascites. On Day-3 patients’ distribution among ascites severity grades showed significant (*p* < 0.05) difference in both groups compared to the respective Day-0 distribution with significant difference in favor of study group. Two patients in control group with marked ascites were admitted to the hospital for paracentesis on Day-4 and Day-5, while the 3rd patient showed gradual resolution to moderate ascites on Day-8. On Day-8, patients’ distribution among ascites severity grades showed significant (*p* < 0.05) difference compared to Day-0 distribution in both groups and in study group compared to Day-8 distribution in control group (Table 7).

**Table 7:** Patients’ distribution among severity grades of ascites determined at Day-3 and Day-8 compared to Day-0 distribution in both groups

Time Ascites severity		Day-0		Day-3		Day-8	
		Control	Study	Control	Study	Control	Study
US detected	Low	6 (25%)	8 (33.3%)	13 (54.2%)^a^	18 (75%)^ab^	17 (70.8%)^a^	21 (83.3%)^ab^
	Moderate	18 (75%)	16 (66.7%)	8 (33.3%)	6 (25%)	5 (20.8%)	3 (16.7%)
Marked (Clinically detected)		0	0	3 (12.5%)	0	2 (8.4%)	0

Data are presented as numbers (%); ^a^: significant difference versus respective Day-0 data; ^b^: significant difference versus control group

Five patients (20.9%) required hospitalization; 4 patients in control group and one patient in the study group with non-significantly (*p* > 0.05) lower frequency of hospitalization in the study group. Two patients had severe distension and one had severe nausea and vomiting. These three patients received ondansetron antiemetic therapy in dose of 40 mg ampoule twice daily and crystalloid replacement fluid therapy. No other complications were encountered in patients of both groups.

There was positive significant (*p* < 0.05) correlation between Day-3 serum E2 level and clinical and other laboratory findings and ascites grading in the study group determined on Day-3. On contrary, Day-3 MOD showed non-significant (*p* > 0.05) positive correlation with these parameters and with Day-3 serum E2 levels.

## Discussion

Both therapeutic policies did favorably for patients received GnRH-a protocol and were at high-risk for development of OHSS. Such effect was manifested as reduction of hospitalization rate down to 16.7%; thus establishing the feasibility of outpatient management of such cases and abolished the development of marked ascites that was developed in three patients in control group and required paracentesis to manage two cases.

Despite the favorable outcome of both groups, addition of cetrotide sc injection for three days to embryo freezing improved outcome of patients in the study group manifested as significant reduction of serum E2 and pain scores on daily assessment with significant reduction of frequency and severity of GI manifestations and ascites on Day-3 compared to patients received embryo freezing only.

In line with the effectiveness of GnRH antagonist (cetrotide) therapy for control of frequency and severity of OHSS in high-risk women received GnRH-a induction protocol; Bonilla-Musoles et al. [16] reported that compared to controls, treatment with 3 mg cetrotide seems to be effective in the management of severe OHSS with significantly dropped E2 levels and faster peritoneal fluid regression as measured by US few days after treatment, and none of cetrorelix treated patients required paracentesis.

Hosseini et al. [17] reported that the frequency of moderate and severe OHSS, hospitalization or acute care for OHSS and ascites tap rates were significantly lower with significantly higher patients' satisfaction with cetrotide than in control group and concluded that GnRH antagonist cetrotide seems to be an effective outpatient treatment with rapid onset activity and minimal side effects for the management of early OHSS.

Lainas et al. [18] reported that low-dose luteal GnRH-ant administration in women with severe early OHSS is associated with significantly decreased ovarian volume, ascites, hematocrit, white blood cell count, serum estradiol and progesterone by the end of the day 11 post-oocyte retrieval, indicating rapid resolution of the severe OHSS. Thereafter, Lainas et al. [19] reported improvement of ultrasound and laboratory parameters, indicating regression of severe OHSS, 7-days after start of GnRH antagonist therapy. Recently, Hebisha et al., [20] documented that GnRH antagonist administration on the day of hCG in cases undergoing IVF/ICSI with long agonist protocol is effective in protection of OHSS and does not affect the clinical pregnancy rate or live birth rate.

The current study detected a relation between the results of sequential estimation of serum E2 and outcome manifested as scorings of pain, associated GI manifestations and severity of ascites. Interestingly, sequential estimation of maximal ovarian diameter did not show significant reduction in either group or relation with these parameters. Such discrepancy between serum E2 levels and ovarian maximal diameter may be attributed to the multiplicity of mechanisms underlying development and progression of OHSS and so for its regression. Similarly, Jakimiuk et al. [21] documented that OHSS is still a difficult diagnostic and therapeutic problem, more studies are required to elucidate pathophysiology of OHSS and because of still unknown etiology treatment is empirical.

In support of the multiplicity of underlying pathophysiological mechanisms for development of OHSS, multiple experimental and clinical trials are still trying to explore these mechanisms. Experimentally, Bar-Joseph et al. [22] demonstrated that pigment epithelium-derived factor (PEDF), a novel intrinsic antioxidant of granulosa cells, possesses potent physiologic anti-angiogenic activity that negates VEGF activity and Chuderland et al. [23] shown that expression of PEDF in granulosa cell line is regulated by hCG, reciprocally to VEGF, and that the PEDF-VEGF balance is impaired in OHSS. Thereafter, Miller et al. [24] using rat model found the severity of OHSS is correlated with hCG-induced PEDF-VEGF ovarian expression impaired ratio.

In 2016, Bar-Joseph et al. [25] found that hCG-induced PEDF down-regulation and VEGF up-regulation are mediated by signaling cascades, as protein kinase A, protein kinase C, epidermal growth factor receptor, which emphasizes the delicate regulation of ovarian angiogenesis. Lunger et al. [26] reported the presence of main opiate receptor-1 (OPRM1) on human granulosa cells and found its blocking significantly reduced granulosa cell-derived VEGF levels in granulosa-luteal cells; thus raising a possibility of disturbed ovarian levels of growth factors as basis for development of OHSS; irrespective of the receptor-type for initiation of response.

On the other side, Orvieto et al. [27] provided a firm evidence for the presence of interleukin (IL)-2 and IL-2 mRNA expression in luteinized granulosa cells of the hyperstimulated human ovaries and suggested that IL-2 could activate the systemic inflammatory response characteristic of OHSS. Taskin et al. [28] detected significantly lower ovarian weight, estrogen levels and corpus luteum counts with lower IL-6 intensity and VEGF expression in GnRH antagonist treated animals than in OHSS animals and attributed development of OHSS to increased expression of VEGF secondary to increased IL-6 intensity; thus raising a possibility of inflammatory basis for development of OHSS.

Clinically, Lainas et al. [19] reported significant decline of VEGF 7-days after start of GnRH antagonist therapy and this decline was associated with an improvement of ultrasound and laboratory parameters, indicating regression of severe OHSS.

Delabaere et al. [29] and Langroudi et al. [30] reported spontaneous OHSS during pregnancy in a context of moderate hypothyroidism and supposed that the etiology of spontaneous OHSS should seek hypersecretion of glycoprotein hormones (hCG, TSH, FSH and LH) and/or mutation of FSH and LH receptors; thus raising a possibility of endocrinal basis for development of OHSS.

Orkunoglu-Suer et al. [31] using polymerase chain reaction captured a non-synonymous LH CG-single nucleotide polymorphism in two severe OHSS cases, and verified its presence by conventional sequencing; thus supposing underlying genetic basis for development of OHSS. Blumenfeld [32] reported that supra-physiological E2 level in OHSS may increase the growth hormone-binding protein (GH-BP) bio-neutralizing GH and diminishing the resultant insulin-like growth factor levels necessary for optimal synergism with FSH.

## Conclusion

Three-day cetrotide therapy starting after oocyte retrieval with embryo freezing could be considered as an appropriate management policy for women received GnHR-agonist induction protocol and were at high-risk for OHSS development. Sequential E2 serum level estimations could predict outcome perfectly than sequential determination of maximal ovarian diameter.

## Abbreviations

CG: Cytosine guanine
COX-2: Cyclo-oxygenase 2
E2: Estradiol
FSH: Follicle stimulating hormone
GH-BP: Growth hormone-binding protein
GI: Gastrointestinal
GnRH-a: Gonadotropin-releasing hormone agonist
GnRH-ant: Gn-RH-antagonist
hCG: Human chorionic gonadotropin
Ht%: Hematocrite value
ICSI: Intra-cytoplasmic sperm injection
IL: Interleukin
IVF: In-vitro fertilization
KSA: Kingdom of Saudi Arabia
LH: Luteinizing hormone
MHz: Megahertz
MOD: Maximal ovarian diameter
mRNA: Messenger ribonucleic acid
OHSS: Ovarian hyperstimulation syndrome
PEDF: Pigment epithelium-derived factor
TLC: Total leucocytic count
TSH: Thyroid stimulating hormone
US: Ultrasound
VAS: Visual analogue scale
VEGF: Vascular endothelial growth factor
